# Outcomes after Acute Malnutrition Program Adaptations to COVID-19, Uganda, Ethiopia, and Somalia

**DOI:** 10.3201/eid2813.212266

**Published:** 2022-12

**Authors:** Talya Shragai, Leisel Talley, Aimee Summers, Hannah Behringer, Maria Wrabel, Heather Stobaugh, Action Against Hunger USA Research Field Team–Ethiopia, Action Against Hunger USA Research Field Team–Somalia, Action Against Hunger USA Research Field Team–Uganda, Eva Leidman

**Affiliations:** Centers for Disease Control and Prevention, Atlanta, Georgia, USA (T. Shragai, L. Talley, A. Summers, E. Leidman);; Emory University, Atlanta (H. Behringer) Action Against Hunger, New York, New York, USA (M. Wrabel, H. Stobaugh);; Tufts University, Boston, Massachusetts, USA (H. Stobaugh)

**Keywords:** COVID-19, 2019 novel coronavirus disease, coronavirus disease, severe acute respiratory syndrome coronavirus 2, SARS-CoV-2, viruses, respiratory infections, zoonoses, childhood malnutrition, community-based management of acute malnutrition, Uganda, Ethiopia, Somalia

## Abstract

At the onset of the COVID-19 pandemic, protocols for community-based management of acute malnutrition (CMAM) were implemented to support continuity of essential feeding services while mitigating COVID-19 transmission. To assess correlations between adaptation timing and CMAM program indicators, we evaluated routine program data in Uganda, Ethiopia, and Somalia for children 6–59 months of age. We specifically analyzed facility-level changes in total admissions, average length of stay (ALOS), total children screened for admission, and recovery rates before and after adaptations. We found no statistically significant changes in program indicators after adaptations. For Somalia, we also analyzed child-level changes in ALOS and in weight and mid–upper arm circumference at admission and discharge. ALOS significantly increased immediately after adaptations and then decreased to preadaptation levels. We found no meaningful changes in either weight or mid–upper arm circumference at admission or discharge. These findings indicate that adapted CMAM programs can remain effective.

In 2020, severe acute malnutrition affected 13.6 million children <5 years of age (*1*), and those affected by severe acute malnutrition were 11.6 times more likely to die than those not affected (*2*). Community-based management of acute malnutrition (CMAM), a proven approach to treat undernutrition, comprises community outreach as well as outpatient and inpatient treatment programs for children with severe acute malnutrition and severe acute malnutrition with medical complications and targets supplementary feeding programs for children with moderate acute malnutrition (*3*). CMAM programs are operational in ≈70 countries worldwide (*4*).

After the COVID-19 pandemic was declared in early 2020, food insecurity was projected to affect childhood nutrition (*5*,*6*). To maintain essential services while mitigating transmission risk, the United Nations Children’s Fund, the Global Nutrition Cluster, the Global Nutrition Cluster Technical Alliance, and the World Health Organization released guidance on CMAM operations during COVID-19 (*7*–*9*). Guidance included adapting normal CMAM protocols to reduce clinic visit frequencies and physical contact between staff and patients; adaptations included longer intervals between clinic visits, training of caregivers to measure the mid–upper arm circumference (MUAC) of their own child and self-refer as needed, and MUAC-only programming. Components of these adaptations have been evaluated in trials and controlled studies (*10*–*12*); however, neither the effect on nutrition outcomes of implementing multiple adaptations at scale nor the effect of all adaptations when implemented by routine programs outside the quality controls of 2-armed cohort trials have been evaluated (*10*).

Given the urgency posed by the COVID-19 pandemic, the adaptations were implemented by CMAM programs despite limited evidence regarding effectiveness (*13*–*17*). To provide information for CMAM programming, we evaluated changes in enrollment and treatment outcome indicators corresponding with implementation of program adaptations for COVID-19.

## Methods

### CMAM Program Data

We asked all outpatient therapeutic programs (OTPs) in Somalia and Ethiopia and all targeted supplemental feeding programs (TSFPs) in Uganda supported by Action Against Hunger USA for children 6–59 months of age to provide electronic data for all dates for which historical data were available via a secure file-sharing platform. Data for all countries ended in December 2020, and for Uganda, data began in January 2019; for Ethiopia, in July 2019; for Somalia at the facility level, in November 2019; and for Somalia at the child level, in January 2017. Analyses included facility-level (outpatient community clinics) indicators of enrollment and treatment outcomes for all 3 countries and child-level indicators for Somalia (Table 1). Program coordinators in each country provided information on the timing and type of protocol adaptations through a separate online survey conducted in July 2020 (*13*,*18*).

**Table 1:** Summary of analyzed CMAM program data and program adaptations implemented by each included program*

Country, level	Program type	No. facilities providing data	Dates data available; date adaptations began	Program outcome variables	Program adaptations
Uganda, facility	Targeted supplementary feeding program	5	Jan 2019–Dec 2020; Apr 2020	Total admissions, recovery rate	Family MUAC, suspension of community screening, reduced frequency of follow-up visits, modified admission/discharge criteria
Ethiopia (Oromia region), facility	Outpatient therapeutic program	81	Jul 2019–Dec 2020; May 2020	Total admissions, recovery rate	Family MUAC, suspension of community screening, reduced frequency of follow-up visits
Somalia					
Facility	Outpatient therapeutic program	12	Nov 2019–Dec 2020; Mar 2020	Total admissions, recovery rate, total screened, average length of stay	Family MUAC circumference, suspension of community screening, reduced frequency of follow-up visits
Child	Outpatient therapeutic program	8	Jan 2017–Nov 2020; Mar 2020	Average length of stay, admission/discharge weight, admission/discharge MUAC	

*Family MUAC trains caregivers to identify childhood malnutrition by using a MUAC tape. Suspension of community screening suspends case finding of childhood malnutrition by community healthcare workers. Reduced frequency of follow-up visits changes the clinic visit schedule for children enrolled in outpatient therapeutic programs and targeted supplemental feeding programs. Modified admission/discharge criteria alter the clinical benchmarks for children to be admitted to/discharged from CMAM programs. CMAM, community-based management of acute malnutrition; MUAC, mid–upper arm circumference.

We evaluated several measures reported monthly by facilities, including total persons screened and total admitted, as well as 2 measures of treatment outcomes: recovery rate and average length of stay (ALOS). Total screened included the number of children for whom MUAC, weight and height, or both were measured at the facility or in the community to assess whether they were malnourished and eligible for admission. Total admissions included all children newly enrolled each month. ALOS was defined as the average number of days elapsed between admission and discharge for all children discharged as recovered, and recovery rate was defined as the percentage of children discharged from the treatment program meeting the discharge criteria by MUAC or weight-for-height z-score.

In Somalia, selected additional indicators were available for all children admitted into OTPs; indicators included length of stay (days) and anthropometric measurements. For each child, weight (kilograms) and MUAC (centimeters) were measured at admission and discharge. For a sensitivity analysis, we compared models testing weight with models testing weight-for-height z-score and weight-for-age z-score.

During the study period, several facilities experienced closures and stock outages. We excluded from analysis all outcomes for months when facilities did not have any children enrolled, and we did not calculate recovery rates for months when children were discharged en masse because of closures or stock outages. For months when a data point from some facilities was missing, we calculated aggregate or mean values for all remaining facilities.

### Covariates

To account for typical increases in enrollment in CMAM programs during the seasonal period of increased food insecurity (lean season), we adjusted models for the timing of the lean season in each country. Similarly, to account for anticipated declines in care-seeking associated with national COVID-19 restrictions, we included as covariates in our models indicators of domestic lockdowns or travel restrictions to assess independent associations with measured program indicators. We extracted data for the period of the lean season in each country from Famine Early Warning Systems Network reports (*19*) and data on COVID-19 mitigation measures data from the Mitigation Tracker maintained internally by the Centers for Disease Control and Prevention (CDC). The Mitigation Tracker database was populated with data from reports and websites from the respective governments and United Nations agencies and media reports, shared with CDC or posted online. We coded COVID-19 mitigation measures and lean seasons as binary variables. We considered as an additional covariate confirmed COVID-19 cases/month but did not include it in the study because of variations in testing policy and surveillance sensitivity in each country.

### Analyses

We constructed interrupted time series models to analyze CMAM program indicator data at the facility and child levels before and after protocol adaptations were put into place while accounting for lean seasons and COVID-19 mitigation measures. We analyzed indicators in individual linear segmented regression models for each country and each indicator.

We aggregated program indicators to the country level after data cleaning and modeled monthly admissions, total children screened, and ALOS as means. Means used as indicators were normally distributed and were robust to differences in the number of reporting facilities per country. We analyzed recovery rates as aggregate rates across all facilities per country.

Models included as fixed effects time, level, and trend changes since protocol adaptations; lean seasons; and COVID-19 mitigation measures. Child-level models also included a random effect for facilities to account for correlation between children in a given facility and baseline differences in indicator values between facilities. Because availability of prepandemic data differed by facility and by country, child-level models included data for all available dates for each facility that provided child-level data.

After the initial adaptations were made, Somalia further adapted protocol. For a sensitivity analysis, outcomes were first modeled with just the initial date of change and then with subsequent dates of protocol change, and results were compared. Models presented include only the initial date of program adaptations.

Two indicators (recovery rate and ALOS) were calculated at discharge and were therefore theorized not to be modified immediately after implementation of program adaptations. Models presented include no time lag in recorded recovery rates and ALOS; however, given an observed median length of stay of 42 (interquartile range 42–49) days, we conducted a sensitivity analysis using time lag periods of 1 and 2 months between the change in COVID-19 policies and those 2 outcomes (data not shown). All other indicators (total admissions, total children screened, and all child-level indicators) were captured at admission and analyzed with no lag.

We performed all data aggregation, cleaning, and analysis by using R version 4.0.3 (https://www.r-project.org). This activity was reviewed by CDC and was conducted consistent with applicable federal law and CDC policy (45 C.F.R. part 46.102(l)(2), 21 C.F.R. part 56; 42 U.S.C. Sect. 241(d); 5 U.S.C. Sect. 552a; 44 U.S.C. Sect. 3501 et seq).

## Results

Five Uganda TSFP facilities, 81 Ethiopia OTP facilities, and 12 Somalia OTP facilities provided facility-level data. For Ethiopia, we dropped 78 facility months from the analysis of recovery rate because of data quality issues; for the facility months excluded, the tally of the total number discharged did not equal the tally of children discharged as recovered, transferred, defaulted, nonresponsive, and dead. Of the Somalia facilities, 8 provided child-level data for 11,719 children and the remaining 4 provided only facility-level data. For Somalia, we dropped 1 facility month from the analysis of total admissions and of total screened because of a complete facility closure and dropped 14 facility months from the analysis of recovery rate and 13 from the analysis of ALOS because of mass discharges resulting from stock outages.

The Uganda team implemented TSFP adaptations for moderate acute malnutrition treatment on April 2020; adaptations included modifying the frequency of TSFP clinic visits from once every 2 weeks to monthly, adding the family MUAC approach (training caregivers to use a MUAC measuring tape to identify malnutrition in their children), suspending community-based screening, and modifying admission and discharge criteria from an upper MUAC threshold of 12.5 cm to 12.9 cm (Table 1). In May 2020, the team working in the Oromia region of Ethiopia modified the frequency of OTP clinic visits from weekly to once every 2 weeks, suspended community-based screening, and began family MUAC. The Somalia team began adaptations in March 2020, modifying the frequency of OTP clinic visits from weekly to once every 2 weeks, suspending community-based screening, and scaling up the family MUAC approach for outreach screening. However, in May 2020, Somalia facilities reverted to using weight-for-height z-score for admissions, and in September 2020, they returned to their preadaptations follow-up visit schedule.

In Uganda, COVID-19 mitigation measures started in March 2020 and extended through the last date for which CMAM data were available (December 2020); measures included a national curfew, a temporary ban on public transit, and a 14-day national lockdown. In Ethiopia, COVID-19 mitigation measures began March 2020 and were still in place through the last date with available CMAM data (December 2020). In Somalia, COVID-19 mitigation measures started in April 2020 and extended through December 2020 and included a national curfew.

### Facility-Level Data

**Figure 1:**
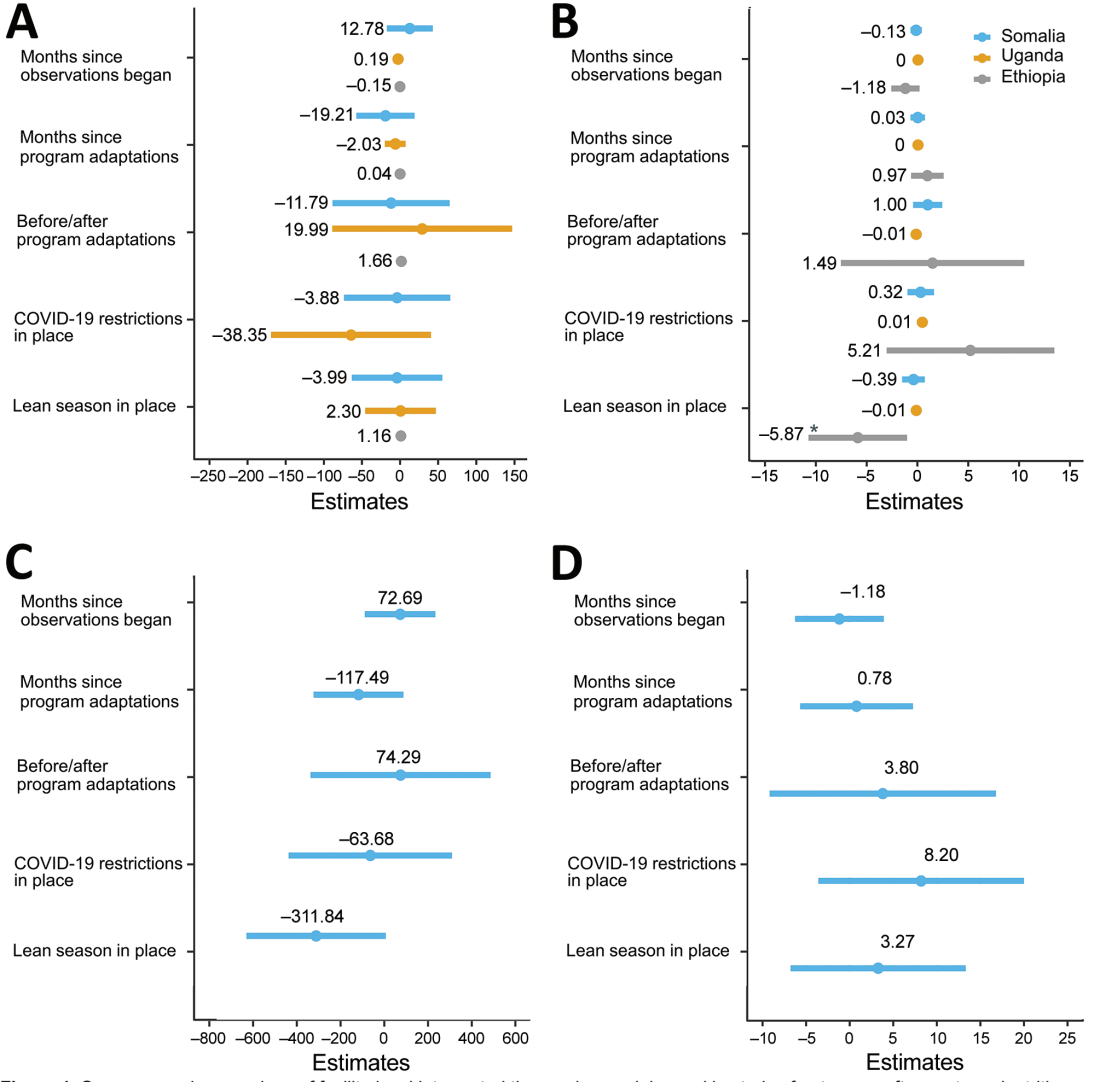
Summary and comparison of facility-level interrupted time series models used in study of outcomes after acute malnutrition programs were adapted for COVID-19 in 3 countries, showing the absolute difference in average total admissions (A), aggregate cure rate (B), average total screened (C), and average length of stay (D) in 12 Somalia outpatient therapeutic facilities, 5 Uganda targeted supplementary feeding program facilities, and 81 Ethiopia outpatient therapeutic program facilities attributed to immediate and long-term effects of program adaptations, lean seasons, and COVID-19 lockdowns. Circles (data markers) and lines indicate point estimates and 95% CIs. Point estimates are labeled, and the asterisk indicates fixed effects with statistically significant results (p<0.05). Total screened and average length of stay was analyzed for Somalia only. COVID-19 restrictions in place refers to COVID-19 mitigation policies that restrict movement, including restrictions on transportation, lockdowns, and curfews. Lean seasons refer to months of increased food insecurity. Time frame analyzed varies by country.

**Figure 2:**
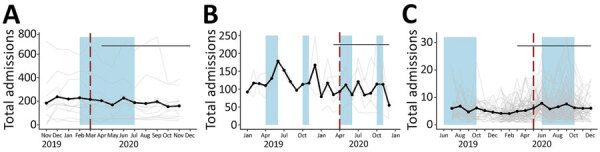
Average total admissions in study of outcomes after acute malnutrition programs were adapted for COVID-19 at 12 Somalia outpatient therapeutic programs (A), 5 Uganda targeted supplementary feeding programs (B), and 81 Ethiopia outpatient therapeutic programs (C) at community management of acute malnutrition facilities. Black dots and lines indicate the mean values across all facilities in each country. Gray lines indicate values for each facility. Red vertical dashed lines indicate dates that program adaptations began. Black horizontal lines indicate dates that COVID-19 restrictions were in place. Blue shading indicates lean seasons. COVID-19 restrictions in place refers to COVID-19 mitigation policies that restrict movement (e.g., restrictions on transportation, lockdowns, and curfews). Lean seasons refer to months of increased food insecurity. Time frame varies for each country.

Mean total admissions in Uganda, Ethiopia, and Somalia did not change significantly after program adaptations (Figure 1, panel A; Figure 2, panels A–C). In the month immediately after revised protocols began, compared with the month immediately before, monthly average total admissions increased in Uganda from 84.6 children to 93.4 children (p = 0.58); in Ethiopia, monthly average admissions increased from 4.4 children to 5.8 children (p = 0.13); and in Somalia, monthly average admissions decreased from 215.7 children to 204.7 children (p = 0.73). The month-to-month trend in mean total admissions after program adaptations compared with the trend in months leading up to program adaptations did not change significantly. In Uganda before adaptations, mean total admissions trended upward at a rate of 0.2 children/month; after adaptations, admissions trended downward by 1.9 children/month (p = 0.62). In Ethiopia, mean total admissions decreased at a rate of 0.2 children/month before protocol adaptations and 0.1 children/month after adaptations (p = 0.87). In Somalia, mean total admissions increased by 12.8 children/month before adaptations and decreased by 6.4 children/month after adaptations (p = 0.28). Our analyses showed no statistically significant effect of lean seasons, COVID-19 lockdowns, or movement restrictions on total admissions (Figure 1, panel A). Results were similar when we included additional variables accounting for subsequent protocol changes for Somalia.

**Figure 3:**
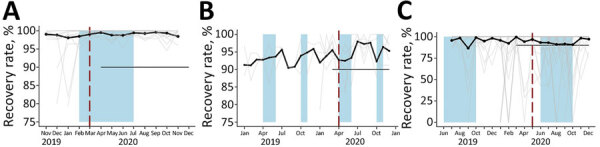
Recovery rates in 12 Somalia outpatient therapeutic programs (A), 5 Uganda targeted supplementary feeding programs (B), and 81 Ethiopia outpatient therapeutic programs (C) at community management of acute malnutrition facilities. Black dots and lines indicate the values across all facilities in each country. Gray lines indicate values for each facility. Red vertical dashed lines indicate date program adaptation began. Black horizontal lines indicate dates that COVID-19 restrictions were in place. Lean seasons are indicated by blue shading. COVID-19 restrictions in place refers to COVID-19 mitigation policies that restrict movement, including restrictions on transportation, lockdowns, and curfews. Lean seasons refer to months of increased food insecurity. Time frame varies for each country.

Similarly, the models showed no significant change in recovery rates after program adaptations (Figure 1, panel B; Figure 3, panels A–C). Recovery rates for all 3 countries were high over the entire period, averaging 93.9% in Uganda, 94.6% in Ethiopia, and 99.0% in Somalia. In the month immediately after program adaptations were implemented, recovery rates dropped by 1.3% in Uganda (p = 0.61), 1.5% in Ethiopia (p = 0.73), and 1.00% in Somalia (p = 0.14); after program adaptations, the monthly trend in recovery rates changed (increased or decreased) by 0.2%/month in Uganda (p = 0.75), 0.8% in Ethiopia (p = 0.21), and 0.03% in Somalia (p = 0.93). However, the recovery rate among CMAM programs in Ethiopia was lower during the lean season, averaging 95.5% outside lean seasons and 92.5% in a lean season (p = 0.022). Results were similar at lags of 1 and 2 months and when additional variables accounting for subsequent protocol changes for Somalia were included.

**Figure 4:**
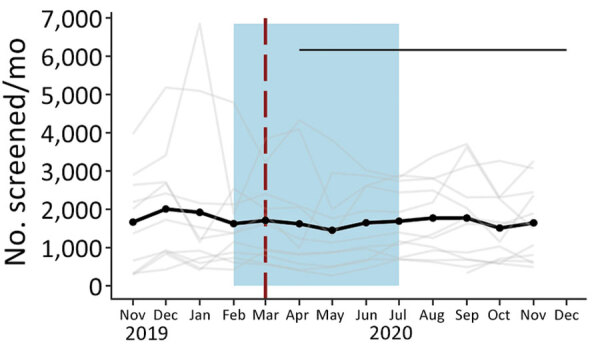
Total screened in community management of acute malnutrition facility outpatient therapeutic programs, Somalia, November 2019–December 2020. Black dots and line indicate the mean values across all facilities. The gray line indicates the raw values for each facility. Red vertical dashed lines indicate date program adaptations began. Black horizontal line indicates dates that COVID-19 restrictions were in place. Blue shading indicates lean seasons. COVID-19 restrictions in place refers to COVID-19 mitigation policies that restrict movement (e.g., restrictions on transportation, lockdowns, and curfews). Lean seasons refer to months of increased food insecurity.

**Figure 5:**
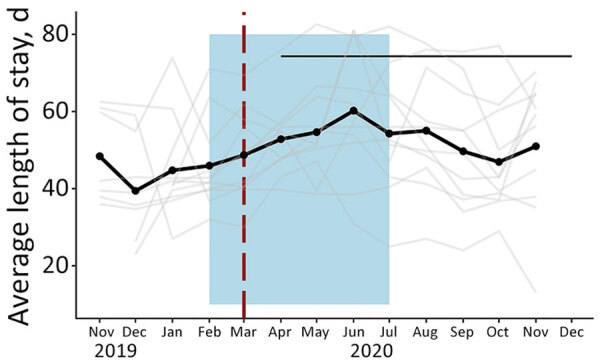
Average length of stay in community management of acute malnutrition facility outpatient therapeutic programs, Somalia, November 2019–December 2020. Black data marker and line indicate the mean value across all facilities. Gray line indicates raw values for each facility. Red vertical dashed lines indicate date program adaptations began. Black horizontal line indicates dates that COVID-19 restrictions were in place. Blue shading indicates lean seasons. COVID-19 restrictions in place refers to COVID-19 mitigation policies that restrict movement (e.g., restrictions on transportation, lockdowns, and curfews). Lean seasons refer to months of increased food insecurity.

Somalia was the only country that provided facility-level data on the total number of children screened and ALOS. Neither outcome indicated a statistically significant change after program adaptations (Figure 1, panels C–D; Figures 4, 5). The average total number of children screened increased from 1,624.8 to 1,708.6 in the month immediately after program adaptations (p = 0.68), and the ALOS increased from 46.0 days to 48.7 days (p = 0.51); neither increase was statistically significant. The rates of total children screened and ALOS were also not statistically significant (p>0.05), and we found no statistically significant effect of either lean season or COVID-19 mitigation measures on either of these outcomes in Somalia. Again, results were similar when we included additional variables accounting for subsequent protocol changes for Somalia.

### Child-Level Data

**Figure 6:**
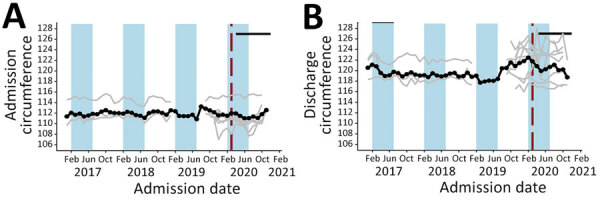
Admission (A) and discharge (B) mid–upper arm circumference at the child level in community management of acute malnutrition facility outpatient therapeutic programs, Somalia, November 2017–November 2020, Black data markers and lines indicate the mean value across all facilities. Gray line indicates raw values for each facility. Red vertical dashed lines indicate date program adaptations began. Black horizontal line indicates dates that COVID-19 restrictions were in place. Blue shading indicates lean seasons. COVID-19 restrictions in place refers to COVID-19 mitigation policies that restrict movement, including restrictions on transportation, lockdowns, and curfews. Lean seasons refer to months of increased food insecurity.

**Figure 7:**
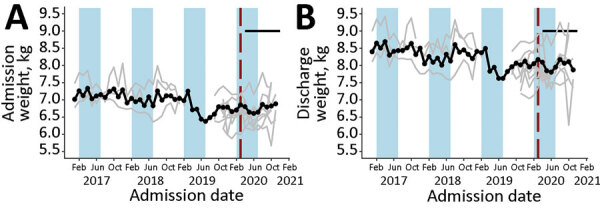
Admission (A) and discharge (B) weight at the child level in community management of acute malnutrition facility outpatient therapeutic programs, Somalia, November 2017–November 2020. Black data markers and line indicate the mean value across all facilities. Gray line indicates raw values for each facility. Red vertical dashed lines indicate date program adaptations began. Black horizontal line indicates dates that COVID-19 restrictions were in place. Blue shading indicates lean seasons. COVID-19 restrictions in place refers to COVID-19 mitigation policies that restrict movement, including restrictions on transportation, lockdowns, and curfews. Lean seasons refer to months of increased food insecurity.

**Figure 8:**
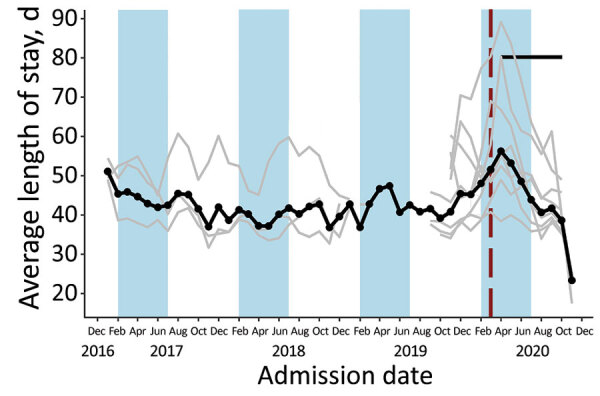
Average length of stay at the child level in community management of acute malnutrition facility outpatient therapeutic programs, Somalia, November 2017–November 2020. Black data markers and line indicate the mean value across all facilities. Gray line indicates raw values for each facility. Red vertical dashed lines indicate date program adaptations began. Black horizontal line indicates dates that COVID-19 restrictions were in place. Blue shading indicates lean seasons. COVID-19 restrictions in place refers to COVID-19 mitigation policies that restrict movement, including restrictions on transportation, lockdowns, and curfews. Lean seasons refer to months of increased food insecurity.

Eight OTP facilities in Somalia provided data through November 2020 at the individual child level. Three facilities provided data from January 2017, four from October 2019, and one from November 2019. From January 2019 through December 2020, the average weight of children at admission was 6.9 kg and at discharge 8.2 kg; the average MUAC at admission was 111.8 cm and at discharge 120.6 cm. None of these metrics changed significantly immediately after program adaptations were implemented (p>0.05 for all comparisons). Changes in trends after adaptations were not statistically significant for MUAC at admission or weight at discharge (Table 2; Figures 6,7). However, trend in MUAC at discharge changed significantly (p = 0.050), as did weight at admission (p = 0.013), although the effect sizes of both were not clinically relevant. Sensitivity analyses comparing admission weight-for-height and weight-for-age z-scores to admission weight showed similar results between all 3 outcomes (data not shown). MUAC at discharge trended upward at a rate of 0.008 mm/month before program adaptations and switched to trend downward at a rate of 0.2 mm/month after program adaptations; weight at admission decreased by 0.01 kg/month before adaptations and increased by 0.04 kg/month afterward (Table 2; Figures 6,7). To put these results into context, before program adaptations, the proportion of children admitted by low weight-for-height only was 11.6%, similar to the 9.3% in the months after program adaptations. Of note, ALOS, which averaged 48.6 days before program adaptations, increased by an average of 12.3 days immediately after program adaptations (p<0.001) and decreased gradually at an average rate of 3.8 days/month, reaching an average of 40.1 days in October 2020 (p<0.001) (Table 2; Figure 8). As for all facility-level results, model outputs were similar for those including variables for subsequent protocol changes.

**Table 2:** Summary of child-level interrupted time-series models showing correlation between program indicators and adaptations, lean seasons, COVID-19 lockdown at 8 Somalia community-based management of acute malnutrition facility outpatient therapeutic programs, Jan 2019 – Nov 2020*

Time in relation to program adaptation	Mid–upper arm circumference at admission, cm			Mid–upper arm circumference at discharge, cm			Weight at admission, kg			Weight at discharge, kg			Average length of stay, d	
	Estimate (95% CI)	p value		Estimate (95% CI)	p value		Estimate (95% CI)	p value		Estimate (95% CI)	p value		Estimate (95% CI)	p value
Months since start	0.0076 (−0.0040 to 0.019)	0.20		0.0082 (−0.019 to 0.036)	0.56		−0.012 (−0.017 to −0.0079)	**<0.001**		−0.014 (−0.020 to −0.0089)	**<0.001**		−0.17 (−0.29 to −0.044)	**0.0084**
Months since program adaptations	0.0038 (−0.093 to 0.10)	0.94		−0.23 (−0.47 to 0.0039)	**0.050**		0.049 (0.011 to 0.087)	**0.013**		0.0077 (−0.040 to 0.055)	0.75		−3.80 (−4.84 to −2.76)	**<0.001**
Before/after program adaptations	0.12 (−0.47 to 0.71)	0.69		0.99 (−0.41 to 2.39)	0.17		0.14 (−0.089 to 0.37)	0.24		0.28 (−0.0017 to 0.57)	0.055		12.30 (5.99 to 18.63)	**<0.001**
COVID-19 mitigations in place	−0.41 (−1.04 to 0.23)	0.21		0.23 (−1.28 to 1.73)	0.77		−0.26 (−0.51 to −0.053)	**0.043**		−0.12 (−0.43 to 0.18)	0.44		9.60 (2.81 to 16.40)	**0.0067**
Lean season in place	−0.26 (−0.51 to −0.017)	**0.039**		−0.20 (−0.79 to 0.38)	0.50		0.044 (−0.14 to 0.053)	0.38		−0.088 (−0.21 to 0.032)	0.16		−0.44 (−3.08 to 2.21)	0.75
Constant	111.26 (110.01 to 112.48)	**<0.001**		120.86 (118.88 to 122.86)	**<0.001**		7.08 (6.82 to 7.34)	**<0.001**		8.44 (8.11 to 8.75)	**<0.001**		53.54 (45.96 to 61.33)	**<0.001**

*Season refers to months of the year with increased food insecurity. COVID-19 mitigations in place refers to months with COVID-19 mitigating lockdowns and/or curfews in place. Boldface indicates statistical significance.

## Discussion

For all 3 countries evaluated, changes in total admissions and total number of children screened after CMAM protocols were adapted for COVID-19 did not differ significantly. Although several facilities temporarily closed because of stock outages, these closures were short term, and after reopening, admissions and total number screened returned to preclosure levels. Modifications to CMAM programs—including family MUAC instead of active case finding by healthcare workers, revising enrollment criteria from either low MUAC or weight-for-height to low MUAC alone, and widening MUAC thresholds—were predicted to affect admissions, but it was not clear if they would cause admissions to rise or fall. Retrospective, observational studies in refugee camps in Cox’s Bazaar, Bangladesh, and in Zambia showed increased total admissions after implementation of family MUAC and reduced frequency of follow-up visits (*14*,*15*). However, those analyses were only descriptive, without quantitative measures of change, limiting comparability. Most likely, the effects depend on context; for example, in select contexts, rising economic and food insecurity may increase underlying prevalence of acute malnutrition.

Our analyses further show no immediate or long-term change in the proportion of children discharged as recovered. Recovery rates were anticipated to deteriorate in programs that decreased the frequency of follow-up visits because the interval between nutritional and medical assessments would be longer, although >1 study has shown that reduced frequency of follow-up visits does not necessarily reduce treatment efficacy (*20*). Furthermore, sharing of rations among siblings, such that the malnourished child receives less than the intended amount, is a known outcome of providing supplementary rations through CMAM programs (*21*,*22*), and anecdotal evidence in multiple contexts reported that when larger portions were distributed to cover longer time intervals between facility visits, sharing and selling of rations increased (*13*), potentially lowering the caloric intake of the child. However, recovery rates across the entire observational period in all countries were well within the global CMAM threshold of >75% recovered recommended by Sphere, a global reference of minimum humanitarian standards (*23*). With the available data, it was not possible to test changes in default rates and nonresponse rates, which remains a topic for future evaluation.

Although the month-to-month trend in admission weight rose significantly after program adaptations, the magnitude of the change—an increase of 0.04 kg/month, or 0.3% of the weight of an average 36-month-old girl—is not programmatically meaningful. Sensitivity analysis accounting for height and age and admission weight also showed no meaningful changes after program adaptations. We may not have observed a meaningful increase in weight at admission because a similar proportion of children were admitted by MUAC only before facilities shifted to all admissions by MUAC only with COVID-19 adaptations. MUAC and weight at discharge, indicators of child profile and health, did not change meaningfully within the Somalia facilities that provided child-level data, consistent with expectations because no adaptations to discharge criteria were adopted. Monitoring future changes in discharge criteria may be useful because >1 study has shown that MUAC-based discharge can result in greater relapse rates (*24*).

Within Somalia, ALOS at the facility level did not change after program adaptations; however, in the subset of Mogadishu-based facilities providing child-level data, ALOS increased by an average of 12.3 days/month, peaking at 60.8 days in April 2020 immediately after program adaptations and then declining. Although the change in program adaptations overlaps with a lean season, potentially confounding the analysis, we found no evidence of such annual pattern in previous years among the 3 facilities providing multiple years of data. A similar immediate increase in ALOS in the month after frequency of follow-up was reduced was also observed in an observational study of OTP data in Nguenyyiel Refugee Settlement in Ethiopia in 2020; however, the program reinstated weekly visits the next month, limiting analysis of longer-term trends (*16*). One other study evaluating ALOS after COVID-19 mitigation adaptations were adopted lacks data from before adaptations were adopted (*14*). Because increasing length of stay can potentially affect programmatic resources, resulting in greater caseloads, higher costs per child, and greater strain on resources as more children stay in programs longer, the need for additional research remains. It is possible that the observed effect of increased ALOS after program adaptations in the Mogadishu region resulted from COVID-19 restrictions implemented in this region, but we were unable to test this hypothesis because of lack of available data. 

The first limitation of this study is that changes in trends observed for several outcomes were not statistically significant; lack of statistical significance may result from insufficient power based on limited time points (Table 1) and high variability of data. Although sample size guidance for interrupted time series models is scarce (*25*), 2 simulation-based power calculations for other interrupted time series designs did not achieve 80% power with 18 time points (*26*,*27*). Given seasonal variation, having >1 year of preintervention data would have been ideal for detecting changes that may be attributed to the COVID-19 protocol adaptations. However, despite limited statistical power, analyzing the magnitude and direction of change of CMAM indicators provides key insight for nutrition programs because the effects of adaptations were previously unknown, and there were multiple, conflicting hypotheses of how programs would be affected. Second, isolating the effects of CMAM program adaptations from other COVID-19 mitigation efforts was challenging because those efforts were implemented around the same time. Third, our data represent a limited number of country experiences; to draw more general conclusions, we would need a larger dataset covering a wide range of countries and program adaptations. Fourth, the lack of country-level average changes in program indicators does not mean that there was no effect in individual facilities. This concept is particularly true in the context of COVID-19, which may have affected use of CMAM facilities in multiple, unpredictable ways. Last, models do not capture the qualitative experience of putting program adaptations into practice. The full context of personnel, environment, and events that shape program success and the challenges facing staff, children, and caretakers are not measured by program indicators and cannot be fully modeled.

Overall, our results suggest that CMAM programs in Uganda, Ethiopia, and Somalia did not undergo consistent, significant changes in program indicators in the first months after adaptations began in response to COVID-19. This finding in turn suggests that CMAM programs may have been able to generally maintain their effectiveness with adapted protocols while continuing to provide service. Although no major or consistent changes were observed after adaptations in these countries in the limited set of indicators considered in this study, it is highly likely that the effects of these program adaptations on program indicators depend on context. Severe acute malnutrition affects 18.7 million children worldwide (*28*), many of whom rely on CMAM programs, so the ability to continue to provide critical services during a pandemic is crucial. As the COVID-19 pandemic has extended over multiple years, programs have experienced protracted staffing shortages and supply chain disruptions. Many of the mitigation measures adapted to reduce transmission may also help alleviate these challenges. Our data provide initial evidence that adaptations to CMAM programs did not significantly affect program efficacy when adopted in the context of the acute onset of the pandemic. However, revisions of global guidance will depend on prospective studies with greater power to evaluate how the revised protocols affect performance outcomes. 
